# Severity of sleep apnea impairs adipose tissue insulin sensitivity in individuals with obesity and newly diagnosed obstructive sleep apnea

**DOI:** 10.3389/frsle.2023.1295301

**Published:** 2023-11-09

**Authors:** Sara Rodrigues, Luiz Aparecido Bortolotto, Robbie A. Beyl, Prachi Singh

**Affiliations:** 1 Pennington Biomedical Research Center, Baton Rouge, LA, United States,; 2 Unidade de Hipertensao, Instituto do Coracao (InCor), Hospital das Clinicas HCFMUSP, Faculdade de Medicina, Universidade de Sao Paulo, São Paulo, Brazil

**Keywords:** obstructive sleep apnea, insulin resistance, free fatty acids, adipose tissue, REM sleep

## Abstract

**Introduction:**

Obstructive sleep apnea (OSA) is a common sleep disorder associated with increased risk for the development of type 2 diabetes. While studies have examined the effects of sleep on whole-body insulin sensitivity, little is known about the effects of sleep on adipose tissue insulin sensitivity in patients with OSA. We analyzed if the severity of OSA, measured by apnea-hypopnea index (AHI), is associated with adipose tissue insulin sensitivity.

**Methods:**

We examined the relationship between sleep parameters and adipose tissue insulin sensitivity in non-diabetic participants with obesity and newly diagnosed OSA who underwent overnight polysomnography and a 2 h oral glucose tolerance test during which circulating free fatty acids were measured. In total, 16 non-diabetic participants with obesity and newly diagnosed OSA (sex, 81.3% males; mean age, 50.9 ± 6.7 y; BMI, 36.5 ± 2.9 kg/m^2^; AHI, 43 ± 20 events/h) were included in the analysis.

**Results:**

In our study participants, AHI is inversely associated with free-fatty acid suppression during oral glucose challenge (*R* = −0.764, *p* = 0.001). This relationship persisted even after statistical adjustment for age (*R* = −0.769, *p* = 0.001), body mass index (*R* = −0.733, *p* = 0.002), waist-to-hip ratio (*R* = −0.741, *p* = 0.004), or percent body fat mass (*R* = −0.0529, *p* = 0.041). Furthermore, whole-body insulin sensitivity as determined by the Matsuda index was associated with percent REM sleep (*R* = 0.552, *p* = 0.027) but not AHI (*R* = −0.119, *p* = 0.660).

**Conclusion:**

In non-diabetic patients with OSA, the severity of sleep apnea is associated with adipose tissue insulin sensitivity but not whole-body insulin sensitivity. The impairments in adipose tissue insulin sensitivity may contribute to the development of type 2 diabetes.

## 1. Introduction

Obstructive sleep apnea (OSA) is a highly prevalent sleep breathing disorder affecting almost 1 billion individuals worldwide (Benjafield et al., 2019). It is characterized by repeated upper airway collapse during sleep which leads to complete (apnea) or partial (hypopnea) cessation of breathing and consequent intermittent exposure to hypoxia, frequent arousals, and sleep fragmentation. Notably, OSA patients are at high risk for development of type 2 diabetes (T2D) independent of obesity (Somers et al., 2008; Pamidi and Tasali, 2012; Drager et al., 2013; Wang et al., 2013; Heinzer et al., 2015; Huang et al., 2018; Strausz et al., 2018). Nevertheless, OSA is highly prevalent in individuals with obesity and both OSA and obesity share similar pathophysiological mechanisms including increased sympathetic tone (Somers et al., 1995), a proinflammatory state, and a dysfunctional glucose metabolism (Reutrakul and Mokhlesi, 2017). Particularly, the rapid eye movement (REM) sleep stage has pronounced sympathetic activity and an important role in glucose metabolism. Studies show that deprivation of REM sleep is associated with impaired sensitivity and release of insulin (Gonnissen et al., 2013).

In recent years, the role of adipose tissue in healthy metabolism and altering insulin sensitivity has been established (Laws et al., 1997). During fasting, adipose tissue provides energy as free fatty acids (FFA) via lipolysis. With food intake, insulin suppresses adipose tissue lipolysis to decrease circulating FFA. When adipose tissue insulin signaling is impaired, FFA may remain elevated for a longer duration. Conversely, increased circulating FFA impairs insulin cellular action to further contribute to insulin resistance via a feed-forward mechanism (Boden, 1999). Insulin secretion and pancreatic beta cell expansion are stimulated by acute exposure to FFA, but prolonged exposure to elevated FFA can inhibit insulin secretion (Haber et al., 2003) and contribute to beta cell apoptosis (Yin et al., 2014) with consequent development of T2D.

Although the FFA are involved in metabolic changes and possibly in the progression of insulin resistance to the development of T2D, little is known about the relationship between OSA, adipose-tissue insulin sensitivity, and the FFA metabolism. In patients with OSA, alterations in FFA metabolism may be potentiated by exposure to intermittent hypoxia and further compounded by the presence of obesity to have a profound effect on adipose tissue and whole-body insulin sensitivity (Ryan, 2017). Indeed, studies show that even in the early stages of insulin resistance, such as in individuals with glucose intolerance; FFA suppression is already reduced (Laws et al., 1997).

In this cross-sectional study, we examine the relationship between adipose tissue insulin sensitivity as determined by FFA suppression during oral glucose challenge and sleep parameters in participants with OSA. We hypothesize that both the severity of hypoxia exposure (AHI) and alteration in sleep architecture via frequent arousals will be associated with adipose tissue and whole-body insulin sensitivity in individuals with OSA.

## 2. Methods

### 2.1. Study population and design

This study uses the baseline data collected for a pilot randomized clinical study including non-diabetic obese OSA participants (NCT04530747). Community advertisements, including targeted and passive outreach (e.g., web, mass emails, community events, etc.), and promotional material were used to recruit study participants from the greater Baton Rouge area. The study was approved by the Pennington Biomedical Research Center (PBRC) Institutional Review Board. All participants provided written informed consent prior to any study procedures.

Eligible participants were between 35 and 65 years old, with a body mass index (BMI) of ≥30 to 50 kg/m^2^, an Apnea-Hypopnea Index of ≥15 events/h, and the ability to provide written informed consent. Participants who met any of the following criteria were excluded: (1) HbA1c > 6.4%; (2) severe or uncontrolled hypertension (systolic ≥ 180 mmHg and/or diastolic blood pressure ≥ 110 mmHg on the average of the three seated measurements after being at rest for at least 5 min); (3) significant cardiovascular, hepatic, renal, neurologic, or psychiatric disease as determined by the study physician; (4) pregnant or breastfeeding; (5) currently taking glucose-lowering or weight loss medications and/or taking antihypertensive and lipid-lowering medications known to affect adipose tissue metabolism; and (6) current or previous use of positive airway pressure (PAP) device in the past 6 months. Eligibility criteria were determined during an in-patient screening visit. If all immediately available eligibility criteria were satisfied, eligible participants were provided oximeters to measure changes in blood oxygen saturation during sleep at home. Participants with an oxygen desaturation index (ODI) >15 events/h of sleep proceeded to the second screening visit during which an overnight sleep study was performed.

The baseline study measures relevant to the current study included DXA for body composition, and a 2 h oral glucose tolerance test for determination of whole body and adipose tissue insulin sensitivity.

### 2.2. Fasting blood draw

A blood sample was collected during the screening visit to assess the inclusion criteria and the participant’s overall health (HbA1c, lipid panel, and glucose), as well as during the baseline study visit to measure insulin, glucose, and FFA.

### 2.3. Overnight sleep study (polysomnography, PSG)

Overnight video PSG was performed in individuals with ODI > 15 events/h. The polysomnography signals were recorded using Grael (Compumedics) and scored using Profusion 4 PSG software by a certified polysomnography technologist. The study included recordings from 9-channel electroencephalography (AASM approved alternate placement), 2-channel electro-oculography, 3-channel electro-cardiography, limb electro-myogram, pulse oximetry, 3 chin electrodes, oronasal airflow by pressure transducer and thermocouple sensors, measurement of respiratory efforts via abdominal and thoracic inductance plethysmography, a microphone, and a body position sensor. Sleep-stage scoring was done on 30-s epochs, and apnea, hypopnea, and arousals were scored using established AASM criteria (Berry, 2020). The sleep study data was verified and interpreted by Sleep Board Certified Physician as well.

### 2.4. Body composition (by dual-energy X-ray absorptiometry)

Dual-energy X-ray absorptiometry (DXA) scan was performed at the Imaging Facility of PBRC. Total adiposity and regional fat mass were assessed using a whole-body scanner (Lunar iDXA; General Electric, Milwaukee, WI). For safety, a pregnancy test was done prior to the scan.

### 2.5. Whole body and adipose tissue insulin sensitivity

Glucose tolerance was assessed using an oral 75 g oral glucose tolerance test (OGTT). Participants were studied after an overnight fast for 12 h. An intravenous line was placed and one baseline sample was collected at −5 min. The participants then consumed a 75 g glucose beverage within 5 min. Blood samples were collected at 30, 60, 90, and 120 min to measure serum glucose, insulin, and free fatty acid concentrations. Whole-body insulin sensitivity was calculated using the Matsuda index [(10,000/fasting glucose × fasting insulin) × (mean OGTT glucose × mean OGTT insulin)] (Matsuda and DeFronzo, 1999). Other calculated indices of whole-body glucose metabolism include the insulinogenic index (measures the early insulin response during the OGTT, Δinsulin/Δglucose from 0 to 30 min) (Goedecke et al., 2009), disposition index (Beta-cell function estimate, Matsuda Index × insulinogenic index) (Utzschneider et al., 2009), and HOMA-IR [(fasting insulin × fasting glucose)/22.5] (Matthews et al., 1985). Adipose tissue insulin sensitivity was calculated using ISI- FFA {2/[Insulin area under the curve (AUC) × FFA AUC] + 1} (Belfiore et al., 2001), Adipo-IR [fasting FFA × fasting Insulin] (Søndergaard et al., 2017), and FFA area over the curve [(FFA0min * 120) − FFA AUC] where AUC was calculated by trapezoid method. FFA suppression as calculated as percentage change [(FFA0min − FFA120min)/FFA0min * 100].

### 2.6. Blood chemistry

Glucose was measured using a Beckman Coulter chemistry analyzer. Insulin was measured by immunoassay (Immulite 2000 Xpi, Seimens). FFA was measured using a commercial colorimetric kit (Wako) using DXC 600 Pro. HbA1c and lipids were determined using a standard automated diagnostic platform. All measures were obtained at the PBRC Clinical Core lab facility.

### 2.7. Statistical analysis

Numerical data are reported as mean ± SD or median (interquartile range), while absolute numbers and percentages are used for categorical variables. The normality of the variable distribution was tested using the Kolmogorov-Smirnov test. Pearson’s correlation determined the relationships between adipose tissue and whole-body insulin sensitivity and the sleep parameters. Variables that presented statistical significance in the bivariate correlation analysis were then introduced in a multiple linear regression analysis to identify independent relationships, with adjustment for age, BMI, % body fat (DEXA), and waist-to-hip ratio (WHR). Data management and statistical analyses were performed using SPSS software (IBM), version 20. For this study, a *p*-value of 0.05 was considered to be significant for the primary outcomes (relationship between FFA suppression %, and Matsuda index with REM % and AHI). Other relationships are considered exploratory.

## 3. Results

In total, 16 non-diabetic participants with obesity and newly diagnosed with OSA (81.3% men; mean age, 50.9 ± 6.7 y; BMI, 36.5 ± 2.9 kg/m 2; AHI, 43 ± 20 events/h) were included in this analysis (Table 1). All participants were abdominally obese as determined by WHR. Only one of the participants had controlled hypertension, and all participants had blood lipids in the normal range. While none of the participants had diabetes, five participants had prediabetes as determined by HbA1c between 5.7 and 5.9%.

Sleep characteristics of the study population are presented in Table 2. As per the study design, all participants had moderate to severe sleep apnea (AHI Range: 23–60 events/h). On average, participants spent only 1.3 [0.6–7.9] % of sleep time in NREM3 and 15.5 ± 8.5% of sleep time in REM sleep stages. Further, the wake after sleep onset of >30 min was observed in 15 participants. In simple correlational analysis, a relationship between fasting FFA, FFA AOC, % FFA suppression during OGTT, and AHI was observed (Table 3). Other parameters of adipose tissue insulin sensitivity were not associated with AHI.

**FIGURE 1:**
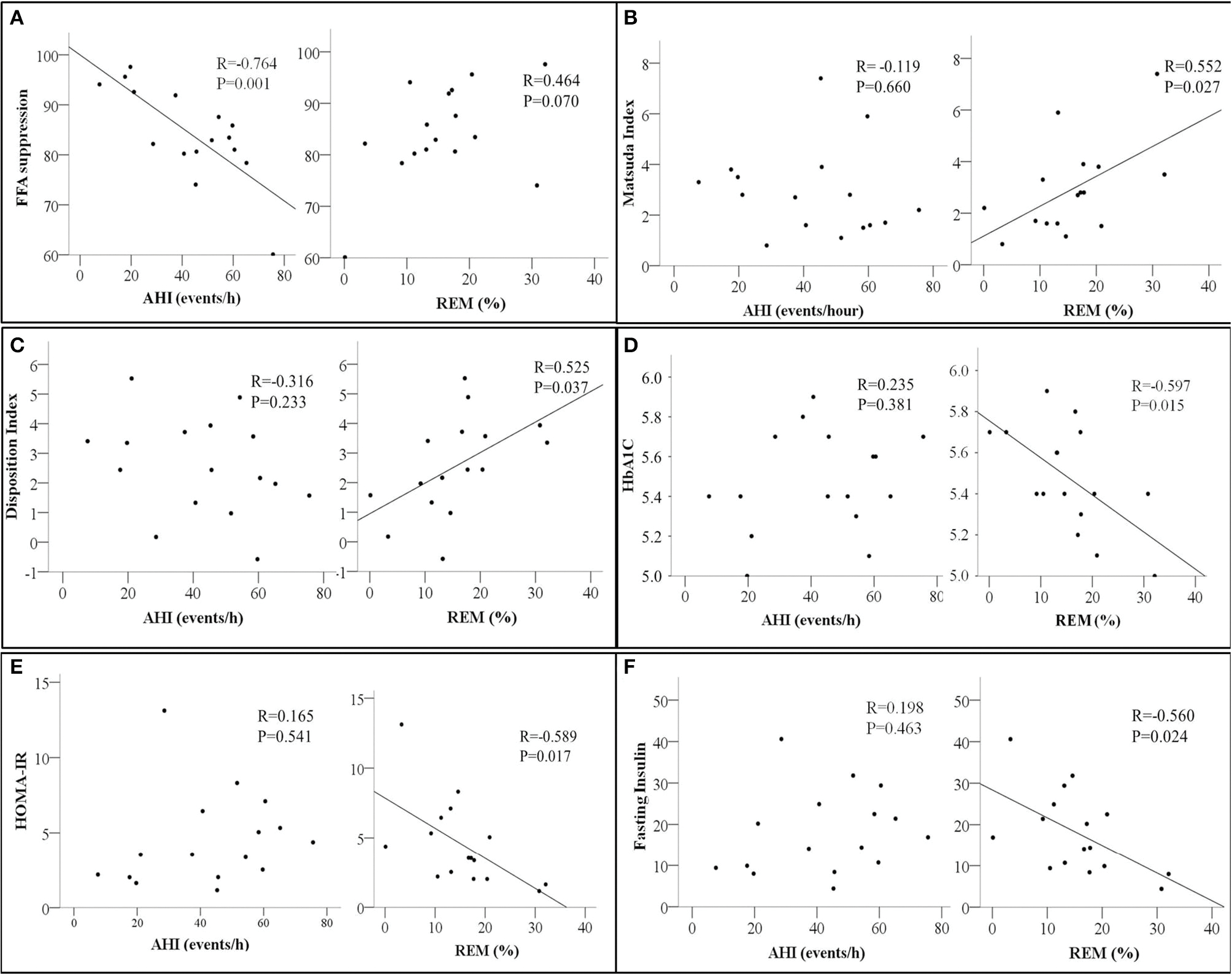
Scatter plots showing the relationship between Apnea Hypopnea Index (AHI- events/hour, left panel) and Rapid Eye Movement Sleep (REM %, right panel) with **(A)** Free Fatty Acids Suppression (FFA suppression %); **(B)** Matsuda index; **(C)** Disposition Index; **(D)** HbA1c, glycated Hemoglobin; **(E)** HOMA-IR, homeostatic model assessment for insulin resistance, and **(F)** Fasting insulin. The correlation coefficient *R* and *p*-values from Pearson’s correlation analysis are presented. *N* = 16.

After adjustment for adiposity-related parameters and age, % FFA suppression remained significantly associated with AHI (Table 3). Percent FFA suppression during OGTT was also inversely associated with REM latency, NREM1 sleep, and arousal index but not REM (Table 4; Figure 1A).

In contrast, measures of whole-body insulin sensitivity such as the Matsuda index, Disposition index, HbA1c, and HOMA-IR were associated with REM sleep but not AHI (Figures 1B–E, respectively). A similar relationship between fasting insulin and REM was also observed (Figure 1F). None of the whole-body and adipose tissue measures of insulin sensitivity and/or resistance were associated with the oxygen desaturation index in our study participants.

**FIGURE 2:**
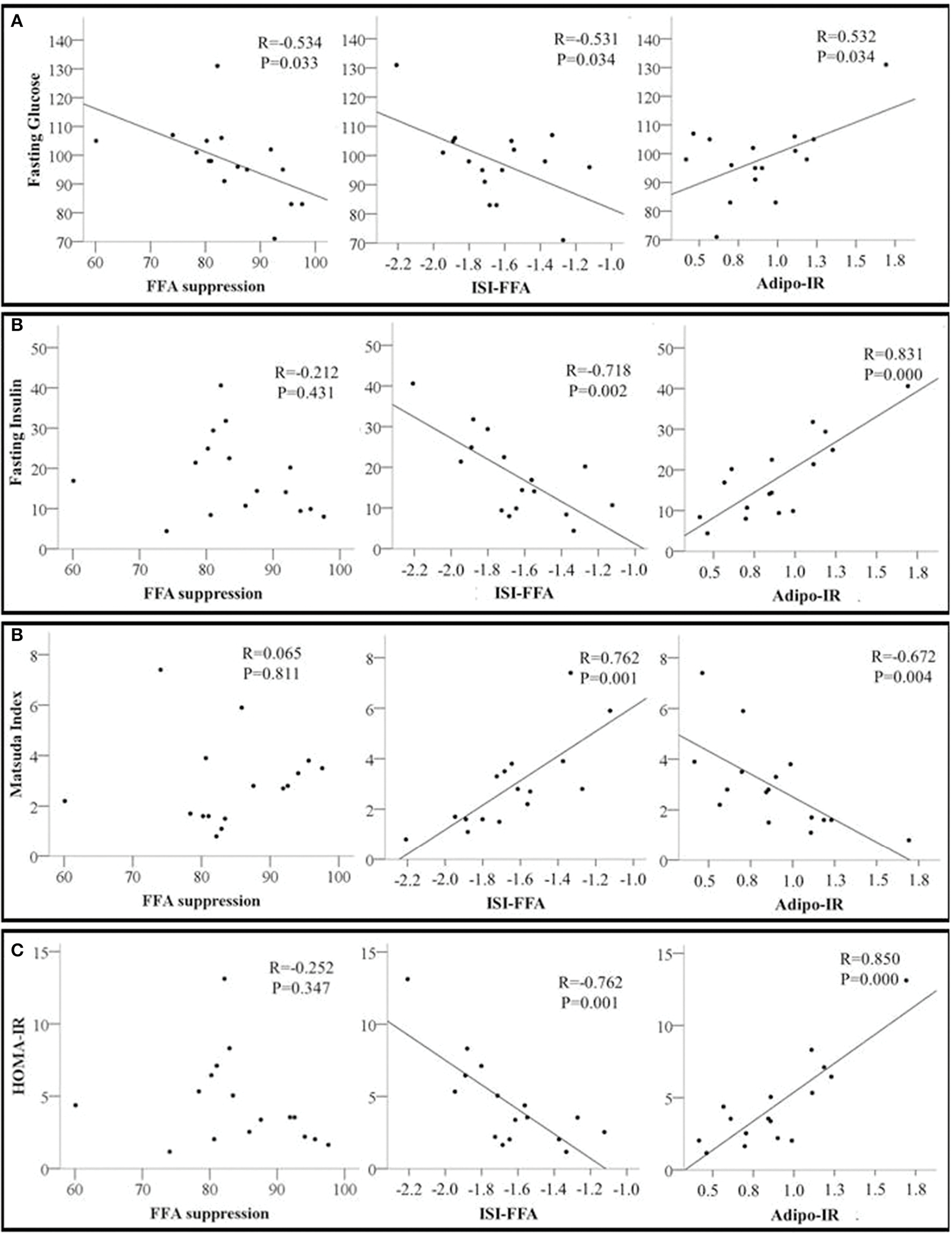
Scatter plots showing the relationship between adipose tissue insulin sensitivity markers including Free Fatty Acids Suppression (FFA suppression %); ISI-FFA, insulin sensitivity index for free fatty acids; Adipo-IR, adipose tissue insulin resistance and whole-body insulin sensitivity markers **(A)** Fasting glucose; **(B)** Fasting insulin; **(C)** Matsuda index; and **(D)** HOMA-IR, homeostatic model assessment for insulin resistance. The correlation coefficient *R* and *p*-values from Pearson’s correlation analysis are presented. *N* = 16.

Next, we examined the relationships between adipose tissue and whole-body insulin sensitivity indices. Percent FFA suppression was inversely associated with fasting glucose but not with any other indices of whole-body insulin sensitivity. ISI-FFA was negatively associated with fasting glucose, insulin, and HOMA-IR but positively associated with the Matsuda Index. Adipo-IR was positively associated with fasting glucose, insulin, and HOMA-IR. An inverse relationship between Adipo-IR and Matsuda index was also observed (Figures 2A–D). Other indices of whole-body insulin sensitivity were not associated with measures of adipose tissue insulin sensitivity (data not shown).

## 4. Discussion

The main finding of the present study is that the severity of sleep apnea is independently associated with adipose tissue insulin sensitivity as determined by percentage FFA suppression, in individuals with obesity and new diagnosis of OSA. Additionally, in our study participants, whole-body insulin sensitivity was associated with REM sleep but not AHI. Together, our study sheds light on the understanding of the relationship between the consequences of OSA on whole-body and adipose tissue-specific glucose metabolism.

Sleep is complexly linked to glucose metabolism (Bopparaju and Surani, 2010; Feng et al., 2021). Both ultradian factors and different sleep stages influence insulin secretion and insulin resistance. Specifically, NREM sleep is associated with insulin resistance while REM sleep is associated with increased cerebral glucose uptake and consequently improved glucose metabolism. A study showed that in healthy men, spikes in glucagon and insulin are more likely to occur during the NREM stage, reflecting the increased secretion of hormones by both alpha and beta pancreatic cells (Kern et al., 1996). The study also showed that a decrease in insulin and glucagon concentrations is apparent in the REM stage. Further, these oscillations seem to be modulated by the central sympathetic nervous system (Kern et al., 1996). Together, the changes in sympathetic activity during different sleep stages seem to alter the secretion of pancreatic hormones which in turn change glucose metabolism.

In patients with OSA, exposure to intermittent hypoxia during sleep and sleep fragmentation are both associated with increased sympathetic activity and may contribute to impaired glucose metabolism (Louis and Punjabi, 2009; Stamatakis and Punjabi, 2010; Gonnissen et al., 2013). When OSA is severe, as seen in our study participants, the frequent apnea/hypopnea-related arousals result in a shortening of the NREM3 and REM sleep durations along with a lengthening of NREM1 and NREM2 sleep duration (Stefanovski et al., 2020). Indeed, compared to the sleep structure of a healthy adult (Kryger et al., 2010), our study participants had an increased NREM1 accompanied by reduced NREM3 and REM sleep. These alterations in sleep structure are therefore likely to contribute to impaired glucose metabolism. Indeed, an inverse relationship between whole-body insulin resistance markers (HbA1c, fasting insulin, and HOMA-IR) and REM sleep was observed in our study. At the same time, indices of insulin sensitivity such as the Matsuda and Disposition indexes were positively associated with REM sleep. It is worth highlighting that the Disposition Index strongly predicts the development of T2D, as it evaluates Beta cell function (Utzschneider et al., 2009). These findings are consistent with a prior experimental study of REM sleep deprivation which showed consequent impaired glucose metabolism (Tasali and Van Cauter, 2002). However, unlike prior studies, we did not observe any relationship between AHI and insulin sensitivity biomarkers such as HOMA-IR and HbA1c (Grimaldi et al., 2014; Chami et al., 2015). The discordant findings may be related to differences in the study population (such as the presence of T2D and absence of OSA in some of the study participants) and our sample size. Unlike these studies, our participants had high AHI with less NREM3 and REM sleep. Furthermore, unlike these studies, we considered AHI in total sleep time, while they found an association between insulin resistance indices only with AHI in REM sleep. Our participants also showed an inverse association between the arousal index (measure of sleep fragmentation) and some indices of insulin sensitivity (whole body-disposition index and adipose tissue-FFA suppression %). The arousal index, also indicative of sympathetic hyperactivity in patients with OSA (Kim et al., 2019), may therefore partly underlie decreases in insulin sensitivity in patients with OSA.

Our results reinforce the relationship between OSA severity and worsening adipose tissue insulin sensitivity shown by Stefanovski et al. (2020). In this study, the authors analyzed overweight and obese individuals divided into groups represented by four quartiles of the AHI according to the severity of OSA. The study showed that adipose insulin resistance was higher in the second and third quartiles compared with the first quartile (with no OSA) and even higher in the fourth quartile, despite multivariable adjustments for age, sex, and DEXA-determined percent body fat. In contrast, our study only included participants with obesity and moderate to severe OSA (AHI > 15 events/hour) and similarly showed an adverse association between the percentage of FFA suppression and AHI. The expected alterations in sleep architecture, namely reduced NREM3 with increases in AHI, were also reported and the participants in the lowest tertile of NREM3 sleep presented a much lower lipolysis suppression rate than those in the highest tertile (Stefanovski et al., 2020). Similarly, our participants exhibited very short NREM3 sleep, as well as longer NREM1, and, despite there being no association between NREM3 and adipose tissue insulin resistance, the NREM1 and REM latency were negatively associated with FFA suppression.

Our findings present intriguing pathways through which OSA treatment may improve insulin sensitivity. As standard PAP therapy is effective in eliminating apnea/hypopnea, this may improve sleep structure and consequently improve whole-body and adipose tissue insulin sensitivity. PAP therapy is likely to play a role in consolidating sleep patterns and promoting NREM3 and REM sleep. Alternate mechanisms through which PAP therapy may improve glucose metabolism would be via a reduction in sympathetic activity. Long-term PAP therapy has been shown to reduce muscle sympathetic traffic in individuals without cardiovascular diseases (Narkiewicz et al., 1999). Together, our study underscores the multifaceted impact of OSA on metabolic processes. However, these mechanisms should be tested in future longitudinal studies. Such studies will help our understanding of the role of PAP therapy in not only managing OSA but also in the prevention of T2D.

This study has several limitations that should be recognized. First, the small sample size used in the study could potentially impact the generalizability of the findings. The absence of a non-OSA control group further confines the interpretation of results, as it prevents meaningful comparisons with a healthy population. Despite these limitations, the results demonstrate a remarkable degree of consistency across the observed data. This internal consistency in results offers valuable information about the relationship between OSA and the role of insulin in FFA metabolism. Further research with larger and more diverse samples, along with controlled comparisons with healthy individuals and in the presence of other cardiovascular diseases, is needed to validate and expand these initial findings.

## 5. Conclusion

OSA severity is associated with adipose tissue insulin sensitivity and can contribute to the development or worsening of insulin resistance via alteration in FFA metabolism. Future studies are needed to examine the causal relationship between sleep fragmentation, AHI, and whole-body and adipose tissue insulin sensitivity to prevent the development of T2D.

## Figures and Tables

**TABLE 1:** Characteristics of the study participants.

**Demographics**	
Age (years)	50.9 ± 6.7
Male/Female	13/3
Weight (Kg)	109.6 ± 14.7
BMI (Kg/m^2^)	36.5 ± 2.9
Body fat (%)	40.2 ± 7.5
Waist (cm)	116 ± 14
Hip (cm)	118 ± 5
Waist-to-hip ratio	1.0 ± 0.1
Waist-to-height ratio	0.7 ± 0.1
SBP (mmHg)	121 ± 13
DBP (mmHg)	81 ± 6
Cholesterol (mg/dL)	213 ± 42
HDL-c (mg/dL)	50 ± 12
LDL-c (mg/dL)	135 ± 32
Triglyceride (mg/dL)	138 ± 65
Non-HDL cholesterol (mg/dL)	163 ± 37
**Whole-body insulin sensitivity**	
HbA1c (%)	5.48 ± 0.25
Fasting glucose (mg/dL)	98 ± 13
Fasting insulin (uU/mL)	18 ± 10
HOMA-IR	4.49 ± 3.10
Matsuda index	2.90 ±1.76
Insulinogenic index	1.02 ± 0.63
Disposition index	2.56 ±1.65
**Adipose tissue insulin sensitivity**	
ISI-FFA	0.02 (0.01–0.04)
Adipo-IR	7.2 (4.3–12.9)
Fasting FFA (mmol/L)	0.57 ±0.27
FFA AOC	33.37 ± 20.93
FFA supression (%)	84 ± 9

Values are presented as mean ± standard deviation or median (interquartile range) based on data distribution (n = 16). BMI, body mass index; SBP, systolic blood pressure; DBP, diastolic blood pressure; HDL-c, high-density lipoprotein cholesterol; LDL-c, low-density lipoprotein cholesterol; HbA1c, glycated Hemoglobin; HOMA-IR, homeostatic model assessment for insulin resistance; ISI-FFA, insulin sensitivity index for free fatty acids; Adipo-IR, adipose tissue insulin resistance; FFA: free fatty acid; AOC, area over the curve during oral glucose tolerance test.

**TABLE 2:** Sleep characteristics of the study population.

Sleep latency (min)	14.5 (3.4–36.5)
REM latency (min)	138.0 (76.8–200.5)
Wake after sleep onset (min)	105.7 ± 68.7
Sleep efficiency (%)	74.5 ± 13.1
NREM1 sleep (%TST)	19.9 ± 7.2
NREM2 sleep (%TST)	60.3 ± 8.3
NREM3 sleep (%TST)	1.3 (0.6–7.9)
REM sleep (%TST)	15.5 ± 8.5
Arousal index (events/h)	37 ± 16
Apnea hypopnea index (events/h)	43 ± 20
REM apnea hypopnea index (events/h)	39 ± 24
NREM apnea hypopnea index (events/h)	42 ± 22
Minimum SpO2 (%)	77.4 ± 7.7
TST with SpO2 < 90 (T90SpO2)	3.3 (1.9–12.9)

Values are presented as mean ± standard deviation or median (interquartile range) based on data distribution. REM, rapid eye movement; NREM, non-rapid eye movement; TST, total sleep time; SPO2, oxygen saturation. N = 16.

**TABLE 3:** Relationship between FFA metabolism and sleep parameters in patients with OSA.

		Standardized regression coefficients		*P*-value	*R*^2^ Adjusted
		B	95% CI for B		
**Fasting FFA**					
Model 1	AHI	−0.525	(−0.014,−0.001)	**0.037**	0.224
Model 2	AHI	−0.139	(−0.011,0.007)	0.643	0.224
	Body fat	0.550	(−0.003, 0.043)	0.084	0.341
Model 3	AHI	−0.484	(−0.014, 0.000)	0.052	0.224
	Age	0.271	(−0.009, 0.031)	0.253	0.247
Model 4	AHI	−0.530	(−0.015, 0.000)	0.051	0.224
	BMI	−0.017	(−0.052, 0.049)	0.946	0.164
Model 5	AHI	−0.648	(−0.017,−0.001)	**0.031**	0.224
	WHR	0.238	(−0.875, 2.098)	0.390	0.212
**FFA AOC**					
Model 1	AHI	−0.661	(−1.105,−0.210)	**0.007**	0.394
Model 2	AHI	−0.338	(−0.928, 0.255)	0.239	0.394
	Body fat	0.460	(−0.351,2.756)	0.117	0.469
Model 3	AHI	−0.605	(−1.015,−0.189)	**0.008**	0.394
	Age	0.375	(−0.117,2.307)	0.073	0.503
Model 4	AHI	−0.694	(−1.174,−0.207)	**0.009**	0.394
	BMI	−0.118	(−4.091,2.491)	0.606	0.358
Model 5	AHI	−0.661	(−1.206,−0.110)	**0.023**	0.394
	WHR	0.000	(−101.013, 101.185)	0.999	0.343
**FFA suppression**					
Model 1	AHI	−0.764	(−0.542,−0.188)	**0.001**	0.554
Model 2	AHI	−0.529	(−0493,−0.012)	**0.041**	0.554
	Body fat	0.335	(−0.211, 1.052)	0.174	0.585
Model 3	AHI	−0.769	(−0.554,−0.181)	**0.001**	0.554
	Age	−0.034	(−0.595, 0.501)	0.855	0.520
Model 4	AHI	−0.733	(−0.540,−0.160)	**0.002**	0.554
	BMI	0.110	(−0.935, 1.649)	0.561	0.532
Model 5	AHI	−0.741	(−0.569,−0.138)	**0.004**	0.554
	WHR	−0.045	(−43.65, 35.77)	0.834	0.521

Values from linear regression analysis are presented. Statistically significant p-values are bolded. FFA, Free Fatty Acids; AHI, Apnea Hypopnea Index; BMI, Body mass index; WHR, Waist to Hip Ratio; AOC, Area over the curve during oral glucose tolerance test.

**TABLE 4:** Relationship between whole-body glucose metabolism and FFA suppression with sleep parameters in OSA patients.

	REM latency (log)	NREM1	NREM2	NREM3 (log)	REM	Arousal index	AHI
HbA1c	0.231 (0.389)	0.239 (0.372)	0.379 (0.148)	−0.200 (0.474)	**−0.597 (0.015)**	0.325 (0.219)	0.235 (0.381)
Fasting insulin	0.230 (0.392)	0.294 (0.269)	0.242 (0.366)	0.295 (0.284)	**−0.560 (0.024)**	0.350 (0.184)	0.198 (0.463)
HOMA-IR	0.363 (0.168)	0.288 (0.280)	0.309 (0.244)	0.216 (0.440)	**−0.589 (0.017)**	0.346 (0.190)	0.165 (0.541)
Matsuda index	0.054 (0.844)	−0.155 (0.567)	−0.292 (0.272)	−0.446 (0.096)	**0.552 (0.027)**	−0.153 (0.573)	−0.119 (0.660)
Disposition index	−0.406 (0.119)	−0.229 (0.395)	−0.309 (0.245)	0.010 (0.971)	**0.525 (0.037)**	**−0.618 (0.011)**	−0.316 (0.233)
FFA suppression	**−0.515 (0.041)**	**−0.743 (0.001)**	0.007 (0.980)	0.282 (0.309)	0.464 (0.070)	**−0.798 (0.000)**	**−0.764 (0.001)**

The correlation coefficient R and p-value (in parenthesis) calculated from Pearson’s correlation analysis are presented. Statistically significant p-values are bolded. HbA1c, glycated Hemoglobin; HOMA-IR, homeostatic model assessment for insulin resistance; FFA, Free FattyAcids; AHI, Apnea Hypopnea Index; REM, Rapid Eye Movement; NREM, Non-Rapid Eye Movement.

## Data Availability

The raw data supporting the conclusions of this article will be made available by the authors, without undue reservation.
